# Pattern of Uveitis in Iran: A Systematic Review

**DOI:** 10.18502/jovr.v16i1.8255

**Published:** 2021-01-20

**Authors:** Masood Bagheri, Mohammad-Hosein Ahoor, Ahad Jafari, Hesam Sadat Hashemi, Mehdi Mohammadkhani

**Affiliations:** ^1^Department of Ophthalmology, Imam Khomeini Eye Center, Kermanshah University of Medical Sciences, Kermanshah, Iran; ^2^Department of Ophthalmology, Nikookari Eye Center, Tabriz University of Medical Sciences, Tabriz, Iran

**Keywords:** Epidemiology, Iran, Systematic Review, Uveitis

## Abstract

**Purpose:**

Uveitis is the third leading cause of blindness worldwide. This study aimed to summarize the pattern of uveitis in Iran through a systematic review.

**Methods:**

This review was conducted according to the guidelines for systematic reviews in the following four steps: literature search, study selection and assessment, inclusion and exclusion criteria, and statistical analysis.

**Results:**

One hundred and fifteen articles were identified by an encyclopedic literature search, and three independent investigators examined them according to the defined inclusion and exclusion criteria. Eventually, 109 manuscripts were retrieved and six cross-sectional studies covering 3,567 patients were included and reviewed. According to the results, the mean age of patients was 40 years, and sex was not a statistically significant predisposing factor. The most common anatomical pattern of involvement was anterior uveitis, and the prevalence of the other three types of uveitis, including middle, posterior, and pan-uveitis, were almost equal. Overall, the most common etiologies of uveitis in the Iranian population were idiopathic uveitis, toxoplasmosis, Behcet's syndrome, and Fuchs heterochromic iridocyclitis.

**Conclusion:**

This study depicted the pattern of uveitis in the Iranian society; this can help physicians in the diagnostic approach, management, and treatment of patients.

##  INTRODUCTION

Uveitis is an umbrella term that includes a wide spectrum of intraocular inflammatory conditions in which the various parts of the eye may be attacked by the immune system.^[1]^

Uveitis refers to inflammation of the uveal tract (iris, ciliary body, and choroid); however, retina, vitreous body, optic nerve, and sclera may also be involved.^[2]^ The etiology of the disease is categorized into traumatic, infectious, and noninfectious-immunologic causes and masquerade syndromes.^[3,4]^

Noninfectious-immunologic uveitis comprises vision-threatening diseases that can be associated with systemic or ocular autoimmune disease, with specific or unknown etiology.^[5]^

More than two million patients worldwide have uveitis,^[1]^ and it has an estimated incidence of 17–52/100,000 person-years. Approximately 35% of these individuals experience severe visual loss and legal blindness^[2]^ and it is the third leading cause of blindness (approximately 5–10% worldwide).^[1,6,7]^ Intermediate, posterior, and pan-uveitis are responsible for visual disabilities in most of these patients. The most common sight-threatening complications are macular edema, retinal detachment, retinal vasculitis, and optic neuropathy. Other causes include phthisis bulbi, hypotony,^[8]^ band keratopathy, and glaucoma.^[1]^

The prevalence, phenotypic features, and distribution of different types of uveitis depend on genetic and epidemiologic factors such as age, sex, race, geographic and environmental influence, and social habits.^[6,9]^ Uveitis may occur in any age group, from infancy to adulthood, but individuals aged 20–60 years old are more susceptible (the incidence in adults is approximately fivefold of that in children).^[2]^ Global studies have found anterior uveitis to be the most common type of involvement seen in both adults and children, but the underlying etiologies differ; for example, juvenile idiopathic arthritis (JIA)-associated uveitis is more common in children and HLA-B27-associated uveitis predominantly affects young adults.^[9]^

In most studies, male and female patients were equally affected.^[3,10]^ However, some causes are more prevalent in a particular gender; for example, HLA-B27-associated anterior uveitis is more common among male patients,^[2]^ and JIA-associated uveitis and multiple sclerosis (MS)-associated intermediate uveitis are more common in young girls.^[11,12,13]^

The epidemiology of non-infectious uveitis is more dependent on racial rather than regional features.^[14]^ The prevalence of infectious uveitis (estimated at 30–50% of all uveitis cases) and some non-infectious posterior uveitis, such as Behcet's and Vogt-Koyanagi-Harada (VKH) syndrome, is higher in developing countries.^[4,15,16]^ Common infectious causes include toxoplasmosis,^[15,17]^ tuberculosis (TB), onchocerciasis, cysticercosis, leprosy, and leptospirosis.^[2]^ The prevalence of some causes of non-infectious uveitis depends on the regional area: for instance, sarcoidosis in Japan.^[18]^ Behcet's disease in countries along the ancient Silk Road (Iran, Turkey, China, Japan, Saudi Arabia, and Greece),^[9,19]^ and VKH syndrome in Asian or Eurasian countries.^[18]^ Generally, the prevalence of infectious uveitis is lower in developed countries; common causes are herpes virus and toxoplasmosis, while other infections, such as TB and syphilis, are rare.^[4]^

Ocular inflammation embraces a broad range of pathologies, both with respect to its etiology and the anatomical location within the eye. For proper listing of the differential diagnosis, practitioners should survey all important information, such as the anatomical location of involvement, pathology (granulomatous vs non-granulomatous), laterality (unilateral vs bilateral), and chronicity (acute, recurrent, or chronic) of the inflammation.^[4]^ The classification of uveitis helps physicians in the diagnostic approach, management, and treatment of patients.

To date, several classification systems have been proposed that vary according to the anatomical location of involvement (primary site of the inflammation), clinical course, etiology, and histopathology.^[20,21]^ Based on the Standardization of Uveitis Nomenclature Working Group,^[21]^ the anatomical location of involvement is classified into four types as follows: anterior, intermediate, posterior, and pan-uveitis (Table 1). This classification is widely accepted today and is now the standard required for the publication of uveitis studies in peer-reviewed literature.

The etiologic distribution of uveitis varies from region to region and parallels that of many studies that have investigated the pattern of uveitis in different parts of the world. Most of the data in this field are from the US and Europe, and reports from developing countries are limited.^[4,14,22]^ Today, an acceptable number of reports that focus on the epidemiology of uveitis in Iran are available; however, all these studies have been conducted in university-based ophthalmology centers. In this study, we review all the available articles on the epidemiology of uveitis in Iran to discuss novel and interesting data regarding the pattern of the disease.

**Table 1:** Anatomical location of involvement in uveitis based on the Standardization of Uveitis Nomenclature (SUN) Working Group


**Type**	**Primary site of inflammation**	**Includes**
Anterior uveitis	Anterior chamber	Iritis
	Iridocyclitis
	Anterior cyclitis
Intermediate uveitis	Vitreous	Pars planitis
	Posterior cyclitis
	Hyalitis
Posterior uveitis	Retina or choroid	Focal, multifocal, or diffuse choroiditis
	Chorioretinitis
	Retinochoroiditis
	Retinitis
	Neuroretinitis
Panuveitis	Anterior chamber, vitreous, and retina or choroid	

**Table 2:** The characteristics of the included studies for meta-analysis and summarized uveitis pattern in the studies carried out at tertiary ophthalmology referral centers in Iran


**Study**	**First author**	**City (university)**	**Publication date (duration of study) **	**Sample size**	**Mean age (range) **	**Male / female **	**Unilateral / bilateral **	**Ant. uveitis n (%)**	**Int. uveitis n (%)**	**Post. uveitis n (%)**	**Pan-uveitis n (%)**	**Infectious/ non-infectious**	**Granulomatous/ non- granulomatous**
Patterns of Uveitis at a Tertiary Referral Center in Northeastern Iran ^[25]^	Hosseini SM	Eye Research Center, Mashhad University of Medical Sciences, Mashhad, Iran.	2018 (Feb 2013 to Mar 2014)	235	35.75 ± 16.34 (3–82)	94/141	85/150	87 (37)	28 (11.9)	10 (4.25)	110 (46.8)	46/189	32/179 (24 undefined)
Clinical Course of Uveitis in Children in a Tertiary Ophthalmology Center in Northwest Iran^[26]^	Alizadeh Ghavidel L	Department of Ophthalmology, Nikookari Eye Center, Tabriz University of Medical Sciences, Tabriz, Iran.	2017 (2003 to 2015)	243	12.3 ± 4.53 (1–18)	113/130	105/138	73 (30)	146 (60.1)	12 (4.9)	12 (4.9)	28/215	40/203
Demographic and Clinical Features of Pediatric Uveitis at a Tertiary Referral Center in Iran^[27]^	Rahimi M	Department of Ophthalmology, Poustchi Eye Research Center, Shiraz University of Medical Sciences, Shiraz, Iran.	2016 (Jan 2007 to Dec 2013)	54	12.5 ± 5 (2–18)	24/30	31/23	22 (40.7)	18 (33.3)	10 (18.5)	4 (7.5)	10/44	—
Clinical Patterns of Uveitis in an Iranian Tertiary Eye-care Center^[28]^	Kianersi F	Isfahan Eye Research Center, Feiz Eye Hospital, Isfahan University of Medical Sciences, Isfahan, Iran.	2015 (1999 to 2012)	2016	33.76 ± 10.56 (2.5–98)	915/1101	1232/784	865 (42.9)	390 (19.3)	432 (21.42)	329 (16.3)	474/1542	176/1840
Patterns of Uveitis at a Tertiary Referral Center in Southern Iran^[29]^	Rahimi M	Poustchi Eye Research Center and Ophthalmology Department, Shiraz University of Medical Sciences, Shiraz, Iran.	2014 (Jun 2005 to July 2011)	475	30.5 ± 15.4 (5–56)	216/259	292/183	190 (40)	53 (11.1)	133 (28)	99 (20.8)	110/365	52/423
Patterns of uveitis in a tertiary eye care center in Iran^[30]^	Soheilian M	Ocular Inflammatory and Uveitis Service, Ophthalmology Department and Ophthalmic Research Center, Labbafinejad Medical Center, Shaheed Beheshti University of Medical Sciences, Tehran, Iran. Negah Eye Center, Tehran, Iran.	2004 (1997 to 2000)	544	32.3 ± 15.2 (—)	238/306	275/269	209 (38.41)	96 (17.6)	101 (18.6)	138 (25.4)	90/454	79/465
Ant. uveitis: anterior uveitis; Int. uveitis: intermediate uveitis; Post. uveitis: posterior uveitis													

**Table 3:** Common etiologies of uveitis in different types in studies carried out at tertiary ophthalmology referral centers in Iran


**Study (First author)**	**Ant. uveitis (%)**	**Int. uveitis**	**Post. uveitis**	**Pan-uveitis (%)**	**Total (%)**
Hosseini SM, et al (2018)^[25]^	Idiopathic (27.5) > FHI (17.24) > Herpetic Uveitis (13.7) = Seronegative Spondyloarthropathy (13.7) > JIA (4.6)	Idiopathic (60.7) > Behcet's syndrome (10.7) = Seronegative Spondyloarthropathy (10.7) > Sarcoidosis (7.1)	Toxoplasmosis (30) > Serpiginous Choroidopathy (20) > Idiopathic (10) = Herpetic Uveitis (10) = Sarcoidosis (10) = Presumed tuberculosis (10)	Idiopathic (22.72) = Behcet's Syndrome(22.72) = VKH(22.72) = Herpetic Uveitis (6.3) = Presumed tuberculosis (6.3)	Idiopathic (28.5) > Behcet's Syndrome (16.6 > VKH (10.6) > Herpetic Uveitis (21) > Seronegative Spondyloarthropathy (6.8) > FU (6.4)
Rahimi M, et al (2016)^[27]^	Idiopathic (59) > JIA (22.7) > Posner-Schlossman (9) > Herpetic Uveitis (4.5) = ALL-L2 (4.5)	Idiopathic (94.4) > Sarcoidosis (5.6)	Toxoplasmosis (40) = Toxocariasis (40) > Idiopathic (20)	Idiopathic (50) > VKH (25) > Sympathetic Ophthalmia (25)	Idiopathic (62.9) > JIA (9.2) > Toxoplasmosis (7.4) = Toxocariasis (7.4) > Herpetic Uveitis (1.8)
Kianersi F, et al (2016)^[28]^	Idiopathic (50.5) > FHI (32.8) > Herpetic Uveitis (7.6) > Behcet's Syndrome (2.6) > JIA (1.3)	Idiopathic (81.6) > Behcet's Syndrome (6.1) >Multiple Sclerosis (4.1)	Toxoplasmosis (90.7) > Idiopathic (4.7) > Behcet's Syndrome (1.4)	Behcet's Syndrome (48) > Idiopathic (32) > VKH (2.7) > ARN (2.4) = Sarcoidosis (2.4)	Idiopathic (43.9) > Toxoplasmosis (19.3) > FHI (14.1) > Behcet's Syndrome (10.5) > Herpetic Uveitis (3.2)
Rahimi M, et al (2014)^[29]^	Idiopathic (44.2) > FHI (17.8) > Seronegative Spondyloarthropathy (10) > Herpetic Uveitis (7.8) = JIA (7.8)	Idiopathic (92.4)	Toxoplasmosis (42.1) > Behcet's Syndrome (15.7) > ARN (8.2) > VKH (6) > Toxocariasis (4.7)	Behcet's Syndrome (34.3) > VKH (17.1) > Endogenous Endophthalmitis (11.4) > Sympathetic Ophthalmia (3)	Idiopathic (37.9) > Behcet's Syndrome (12.4) > Toxoplasmosis (11.8) > FHI (7.1) > VKH (5.2)
Soheilian M, et al (2004)^[30]^	Idiopathic (52.1) > FHI (17.2) > Seronegative Spondyloarthropathy (10) > JIA (4.8) > Herpetic Uveitis (3.8)	Idiopathic (86.5) > Sarcoidosis (7.3) > Multiple Sclerosis (4.2)	Toxoplasmosis (54.5) > Eales Disease (11.9) > Toxocariasis (10.9) > ARN (8.9) > Serpiginous Choroidopathy (4) = APMPPE (4)	Behcet's Syndrome (34.1) > Idiopathic (22.5) > VKH (15.2) > Multifocal Choroiditis and Panuveitis (10.1) > Sarcoidosis (5.1) = Sympathetic Ophthalmia (5.1)	Idiopathic (45.5) > Toxoplasmosis (10.1) > Behcet's Syndrome (8.6) > FHI (6.6) > VKH (3.9)
Ant. uveitis: anterior uveitis; Int. uveitis: intermediate uveitis; Post. uveitis: posterior uveitis				

##  METHODS

**Figure 1:**
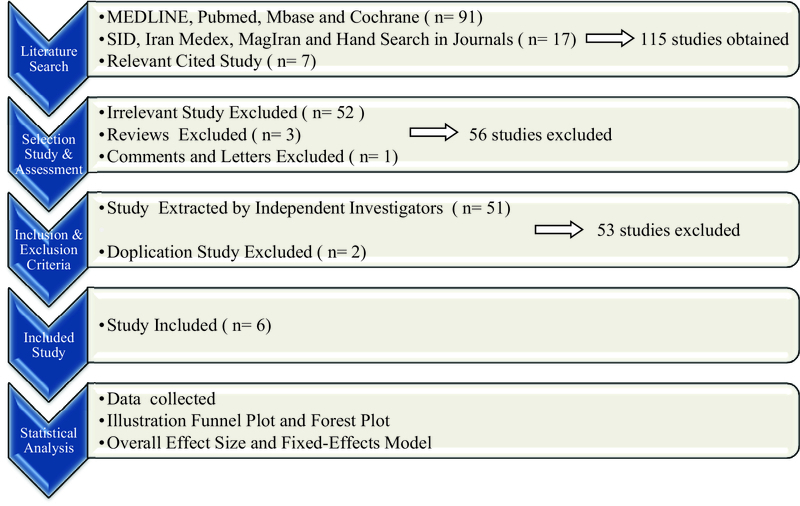
The methodology of the study step by step. During an encyclopedic literature search and survey of relevant cited studies, 115 studies were found, where 56 were excluded in the second step (irrelevant studies, reviews, comments and letters). After reviewing the inclusion and exclusion criteria, 53 more studies were excluded, and finally, 6 studies were included for statistical analysis.

This review was conducted according to the guidelines for systematic reviews in healthcare^[23]^ in four steps as described below (methodology described in Figure 1):

**Figure 2:**
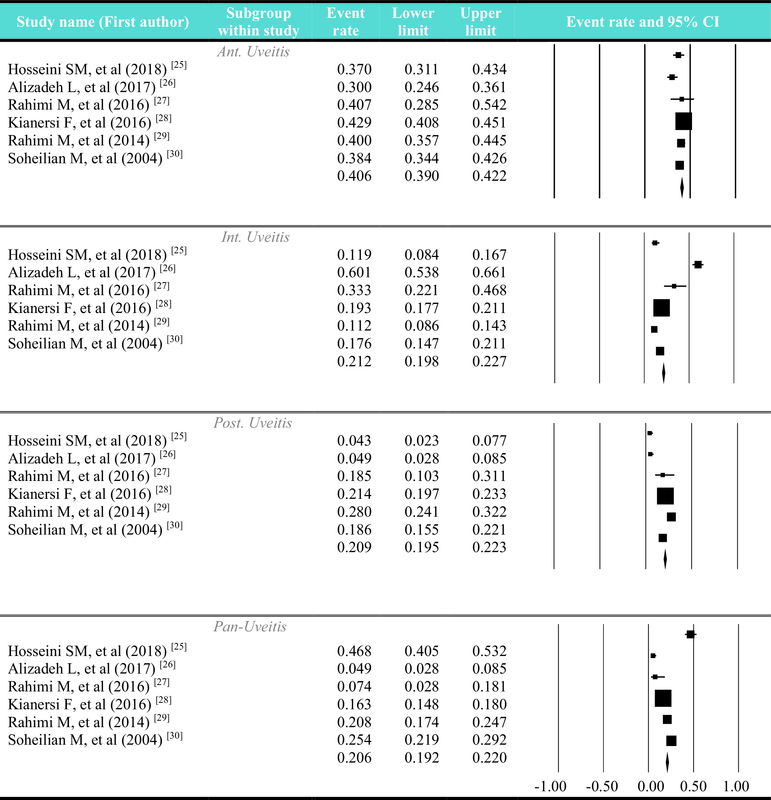
Pattern of uveitis based on anatomical location of involvement explained in this figure according to the studies separately. Ant. uveitis: anterior uveitis; Int. uveitis: intermediate uveitis; Post. uveitis: posterior uveitis

### Literature Search

An encyclopedic literature search for articles published up to July 2019 was conducted on MEDLINE, EMBASE, and the Cochrane library. No language limitations were applied.

All studies that reported the epidemiology of uveitis in Iranian patients were detected based on the medical subject heading (MeSH) terms for the following search strategy:

“{[(“Uveitis” or “Panuveitis” or “Ophthalmia, Sympathetic” or “Uveitis, Anterior” or “Uveitis, Posterior” or “Uveitis, Intermediate” or “Pars Planitis” or “Uveitis, Suppurative” or “Panophthalmitis”).af.] AND (ocular inflammation) AND (iran.mp. [mp=ti, ot, ab, tx, ct, sh, kw, ps, sj, do, dv, po, go, rs, nm, hw, an, ui])}.”

In addition, a broad literature search was conducted using Persian databases such as IranMedex (www.iranmedex.com), Scientific Information Database (www.sid.ir), and MagIran (www.magiran.com). A manual search was performed in the following journals: *Journal of Ophthalmic and Vision Research* (http://www.jovr.org), *Journal of Current Ophthalmology* (https://www.journals.elsevier.com/journal-of-current-ophthalmology), and *Bina Journal of Ophthalmology* (binajournal.org).

Finally, the cited references in the obtained studies were manually reviewed for relevant articles. A total of 15 articles were found in this step.

### Study Selection & Assessment

Articles that were most relevant to our topic were selected, and among them, the reported prevalence, incidence, or epidemiologic pattern of uveitis were thoroughly studied.

### Inclusion & Exclusion Criteria

Two researchers, M.B. (MD, ophthalmologist, vitreoretinal surgeon) and A.J. (MD, general ophthalmologist), independently assessed the titles and abstracts identified in the previous step for potential eligibility, and the full-text articles were retrieved for studies on the epidemiological pattern of uveitis in the Iranian population. Fifty-nine studies were found and all their full-text versions were obtained. To avoid potential bias or errors, three independent individuals, M.B., A.J., and H.S.H. (MD, statisticians) examined the quality of the papers separately according to the checklist for critical appraisal and data extraction for systematic reviews of prediction modelling studies (CHARMS).^[24]^ Then, the data were extracted. Discrepancies were resolved by a consensus or discussion with the fourth reviewer, M.H.A. (MD, ophthalmologist, vitreoretinal surgeon), if necessary. Eventually, six cross-sectional studies covering 3,567 patients and data extracted by the investigators were included, and the final data were matched.

### Statistical Analysis

The following data were collected from each study: the name of the first author, publication date, city or academic center, duration of the study, number of patients, demographic characteristics, anatomical pattern of involvement, etc. (Table 2).

Data were analyzed using the Comprehensive Meta-Analysis.2 (CMA.2) software. The heterogeneity index was assessed using the I${}^{2}$ test. A random-effects model was employed if the test revealed substantial heterogeneity (I${}^{2}$ > 50%). If non-significant (I${}^{2}$ ≤ 50%), a fixed-effects model was used.^[31]^ The level of significance for both heterogeneity and the pooled effect was adjusted at *P* < 0.05.

##  RESULTS

Of the nine studies that examined the epidemiology of uveitis in the Iranian society, three were excluded because two were duplicates^[32,33]^ and one was conducted only in patients with posterior uveitis.^[34]^ Finally, data from six studies were analyzed; two involved cases of pediatric uveitis and four involved adults. Except for two studies that examined pediatric uveitis (patients enrolled in the age range <18 years),^[26,27]^ the mean age of the patients included in the studies was 40 years.^[25,28,29,30]^. In all reports, the disease was more common in women than in men, except in the study by Hosseini *et al*, where this ratio was statistically significant (female to male ratio was 1.5).^[25]^

Statistical analysis showed that the most common anatomical pattern of involvement in the tertiary referral ophthalmology centers was anterior uveitis (event rate: 40.6% among all uveitis patients), but the prevalence of the other three types including middle, posterior, and pan-uveitis was almost equal (because of the non-significant I${}^{2}$, the fixed-effects model was used to estimate the overall effect size; data not shown). In the majority of studies, the most common anatomical site of involvement was anterior uveitis^[27,28,29,30]^ except in the reports by Hosseini *et al* ^[25]^ (pan-uveitis was prevalent in 110 out of 235 involved; 46.8%) and Alizadeh-Ghavidel *et al* ^[26]^ (intermediate uveitis was prevalent with 146 out of 243 involved; 60.1%). The rarest anatomical site of involvement in three studies was pan-uveitis;^[26,27][28]^ however, this was not the case in the reports by Hosseini *et al* ^[25]^ (posterior uveitis was the rarest with 10 out of 235 cases involved; 4.25%), Rahimi *et al*,^[29]^ and Soheilian *et al* ^[30]^ (intermediate uveitis was the rarest with 53 out of 475 cases [11.1%] and 96 out of 544 cases [17.6%], respectively). The study-wise pattern of uveitis based on the anatomical location of involvement has been shown in Figure 2.

In most studies, binocular involvement was more common, but in the studies by Hosseini *et al* and Alizadeh-Ghavidel *et al*, monocular involvement was more prevalent.^[25,26]^ In all studies conducted in ophthalmology referral centers, the most common type of pathological involvement in patients was non-granulomatosis uveitis (compared to the granulomatous type). The prevalence of non-infectious uveitis in all studies was higher than that of infectious uveitis, although in the pattern of posterior uveitis, the infectious type was more common than the non-infectious type due to toxoplasma retinochoroiditis. Table 2 summarizes the uveitis pattern in the studies carried out at tertiary ophthalmology referral centers in Iran.

In the study by Hosseini *et al*, which was conducted at an ophthalmology referral center in north eastern Iran, idiopathic uveitis was more common overall (67 cases of 235; 28.5%) and in different uveitis types, except posterior uveitis in which toxoplasma retinochoroiditis was prevalent (3 cases, 10; 10%). After idiopathic uveitis, Behcet's syndrome (39 patients; 16.6%), VKH (25 patients, 10.6%), herpetic uveitis (21 patients, 8.9%), and seronegative spondyloarthropathy (16 patients, 6.8%)^[25]^ were the other common etiologies in different uveitis types.

In the study by Alizadeh-Ghavidel *et al*, which was conducted at an ophthalmology referral center in the northwest of Iran, idiopathic uveitis was more common overall (117 cases of 243; 48.1%), followed by toxoplasma retinochoroiditis (5.3%).^[26]^

In the study by Kianersi *et al*, conducted at an ophthalmology referral center in Iran, idiopathic uveitis was more common overall (882 cases of 2016; 43.9%), followed by toxoplasma retinochoroiditis (19.3%), Fuchs heterochromic iridocyclitis (FHI) (14.1%), Behcet's syndrome (10.5%), and herpetic uveitis (3.2%).^[28]^

In the study by Rahimi *et al*, which was conducted at an ophthalmology referral center in southern Iran, idiopathic uveitis was more common overall (180 cases of 475; 37.9%). The most common etiologies of idiopathic uveitis were Behcet's syndrome (12.4%), toxoplasma retinochoroiditis (11.8%), FHI (7.1%), and VKH (5.2%).^[29]^

The first study on the epidemiology of uveitis in the Iranian population was reported by Soheilian *et al* in 2004 at a tertiary referral center in Tehran. Similar to other studies, idiopathic uveitis was the most common type of involvement (231 patients out of 544; 45.5%). Other prevalent etiologies in different uveitis types were toxoplasma retinochoroiditis (10.1%), Behcet's syndrome (8.6%), FHI (6.6%), and VKH (3.9%).^[30]^ Table 3 shows the common etiologies of uveitis in different types of studies carried out at tertiary ophthalmology referral centers in Iran.

##  DISCUSSION

Uveitis as a potentially sight-threatening ocular disease poses diagnostic and therapeutic challenges for general ophthalmologists as well as uveitis specialists. Epidemiological studies of the pattern and etiologies of uveitis can help clinicians diagnose, manage, and treat the disease. However, epidemiological studies on the disease at a national level can aid in assessing the burden of the disease on the country's health community, making it possible to plan for the future. In contrast, studies on the incidence and prevalence of uveitis in our society are limited, especially in the general population. Based on the extensive literature review, to the best of our knowledge, no study has reported the epidemiological pattern of uveitis in the general Iranian population, and no study has been conducted in the field of general ophthalmology (all reports were from referral tertiary ophthalmology centers).

The clinical pattern of uveitis may change over time for several reasons such as emerging diseases, new surgical procedures that can lead to uveitis as a complication, and new laboratory equipment that may help to better understand or further diagnose the disease. Certainly, the limitations of laboratory equipment can make it difficult to detect some etiologies and cause some specific diagnosis to fall into the category of idiopathic uveitis. Thus, the pattern of uveitis in one community may be different from that in other societies and may also change over time. This justifies the need for national and regional studies and repeated epidemiological studies over time. Comparison of these studies could help identify the predisposing factors in different regions, provide new insights into the pathogenesis of the disease, and clarify the path for future studies.

In the present study, the mean age of the patients included in the articles reviewed was 40 years, and gender was not a statistically significant predisposing factor. The most common anatomical pattern of involvement was anterior uveitis. However, the prevalence of the other three types including middle, posterior, and pan-uveitis was almost equal. The most common clinical features of the disease were binocular uveitis (compared to the monocular), non-granulomatosis uveitis (compared to the type of granulomatosis), and non-infectious (compared to the infectious) involvement. Overall, the prevalent etiologies were idiopathic uveitis, toxoplasmosis, Behcet's syndrome, and FHI. In the subgroup analysis, the most common etiologies for anterior uveitis were idiopathic uveitis, FHI, and herpetic uveitis; for intermediate uveitis, Behcet's syndrome and MS were common; and for posterior uveitis toxoplasmosis, idiopathic uveitis and Behcet's syndrome were common. In pan-uveitis, Behcet's syndrome, idiopathic uveitis, and VKH syndrome were most prevalent.

All published studies have examined the epidemiology of uveitis in university referral ophthalmology centers. Therefore, the results of this study cannot be generalized to the public because there are significant differences between the pattern of disease in these studies compared to general ophthalmology practice or the community.

Similar to the present study, most worldwide reports have shown that anterior uveitis is the most common type of involvement, followed by pan-uveitis, posterior, and intermediate uveitis.^[1,9,35,36]^ However, most of these studies have been carried out in university referral centers, and their results cannot be applied to the general public. In these settings, a higher proportion of patients with posterior and pan-uveitis and a lower proportion of those with anterior uveitis are expected to be comparable.^[4,37]^

The pattern of uveitis can be influenced by several epidemiologic factors; therefore, any comparison should consider these differences. The regional-based epidemiological studies can be useful for both diagnostic and therapeutic guidance. This may be more important in developing countries such as Iran because of its resource constraints and a higher prevalence of the disease in some uveitic entities (compared to developed countries), and its complications, especially blindness.^[4,22,38,39,40]^

This study has some limitations. First, this study was limited by the inclusion and exclusion criteria of the studies reviewed; for example, all studies considered traumatic uveitis as exclusion criteria, while Das *et al* reported a prevalence of 5%.^[35]^ Second, reports on the epidemiology of uveitis in Iran have covered different time periods that may be difficult to compare. Even in a single community, the pattern of uveitis can change over time for several reasons such as emerging diseases, advances in laboratory equipment, and changes in diagnostic criteria. However, when comparing studies from different cities, some factors such as the socioeconomic level of the region, can change the face of the disease. In under-resourced areas, an underrepresentation of mild or moderate cases of uveitis is expected because of limited access to medical facilities.^[4,22,38,39,40]^ Third, this study was limited by the inclusion of all types of uveitis and different age ranges; considering the heterogeneity in the selected studies and the non-representative population, aggregate estimates for the prevalence of uveitis could not be made in the current review. However, according to the survey in the Iranian population, the heterogeneity of patients in terms of racial factors compared to other global studies was minimal. Finally, the survey of the referral centers may have been influenced by referral bias; therefore, they do not reflect an appropriate view of the disease pattern in society or in general practice. Therefore, subsequent analysis focusing on homogeneous age groups can provide more accurate results regarding the pattern of uveitis. In addition, future epidemiologic studies are recommended in the general population and in the field of general ophthalmology.

##  Financial Support and Sponsorship

Nil.

##  Conflicts of Interest

There are no conflicts of interest.
